# Team approach to osteoarthritis management: Viewpoints of biokineticists and physiotherapists in South Africa

**DOI:** 10.17159/2078-516X/2023/v35i1a15260

**Published:** 2023-05-09

**Authors:** R Gilchrist, A Kholvadia

**Affiliations:** Department of Human Movement Science, Faculty of Health Sciences, Nelson Mandela University, Gqeberha, South Africa, 6001

**Keywords:** biokinetics, chronic disease management, healthcare teams, interprofessional education, physiotherapy

## Abstract

**Background:**

The rehabilitative nature of biokinetics and physiotherapy in osteoarthritis management highlights a complex interaction between different professions to achieve effective outcomes for the patient. The success of a team approach is dependent on key competencies for optimal patient-focused care and appropriate cross-referral systems.

**Objectives:**

To explore and describe the viewpoints of biokineticists and physiotherapists regarding a team approach to osteoarthritis management in the South African public and private healthcare setting.

**Methods:**

A descriptive methodology with a convenience sampling technique was used. The target population consisted of biokineticists (n=47) and physiotherapists (n=165) located within the South African healthcare sectors. A self-administered, online questionnaire surveyed rehabilitative professionals’ views of a team approach to osteoarthritis management.

**Results:**

There is no evidence that the biokineticists and physiotherapists differ with respect to how they rate the communication between team members in osteoarthritis management (p=0.68). Communication was viewed as neither of a high nor low quality by biokineticists (43%) and physiotherapists (36%). Biokineticists (54%) and physiotherapists (69%) felt adequately equipped on their understanding of the role of various healthcare professions involved in osteoarthritis management (p=0.22). However, 43% of rehabilitative professionals indicated that they had not been exposed to interprofessional education initiatives (p=0.61).

**Conclusion:**

Both professions were well-versed on the roles of various professions involved in osteoarthritis management, however, communication was not optimal. While this study creates an awareness of the benefits of team-based management for osteoarthritis, the findings could stimulate debate on the optimal implementation of key competencies required for effective teamwork, thereby facilitating patient-focused care and referral systems.

There are challenges within the South African healthcare system when providing the appropriate continuum of care to address the complexities of patients with osteoarthritis (OA).^[1]^ The burden and complexity of the disease, the influence of poverty, infrastructure challenges, lack of resources and funding for rehabilitation, and staff shortages^[1]^ create a substantial strain on the healthcare platform. Furthermore, collaborative systems in OA have several impediments to care, including restrictions in access to healthcare services, communication deficiencies, unfamiliarity with the roles of healthcare professionals (HCPs), lack of knowledge by HCPs, and inequities in care.^[2]^

Collaborative practice is a typical feature of OA care with treatment provided by a range of allied and medical professionals, and the patients themselves.^[2]^ The scope of practice (SoP) of biokinetics and physiotherapy is ideally suited to OA management^[3]^ as set out by the Health Professions Council of South Africa’s (HPCSA) Professional Board for Physiotherapy, Podiatry, and Biokinetics.^[4]^ The SoP of both professions, as stipulated by the HPCSA, allows for the prescription of rehabilitative exercises.^[4]^ Previous research^[5]^ clarified the overlap in exercise prescription between the two professions by stating how acute symptomatic treatment of chronic conditions, such as OA, involves acute phase mobilisation and manipulation by physiotherapists. The publication mentioned above later stated that if surgery is warranted, the patient may undergo acute phase physiotherapy initially, followed by sub-acute phase physiotherapy to control pain and disability.^[4,5]^ In accordance with recommended clinical guidelines for OA,^[3]^ referral to a biokineticist for final phase functional exercises should then occur at Week six of the rehabilitation plan^[5]^ to assist the patient to return to functional activities of daily living.^[3,6]^ Thus therapeutic exercise and symptom relief treatment modalities offered by biokineticists and physiotherapists are important for optimal OA management,^[3]^ particularly as evidence shows a favourable effect compared with sedentary individuals.^[7–9]^ The above research highlights the indispensable role each profession plays in the management of OA at the different phases of the rehabilitation process.

The therapeutic and rehabilitative nature of both professions at different phases of OA management highlight a complex interaction which emphasises the need for a coordinated approach among different professions to achieve effective outcomes for the patient. This notion is supported by research which indicates that a team of HCPs can effectively deliver optimal patient-focused care,^[8,10]^ which is a holistic approach to individualised care compared to the traditional biomedical model of medicine that regards the patient as a disease carrier requiring diagnosis and treatment.^[8]^ Such holistic care addresses the multidimensional needs of OA patients by allowing the patient to play an active role in their healthcare^[2,11]^ and has the potential to improve the quality of their healthcare.^[2]^ Local researchers ^[5]^ have iterated the effectiveness of healthcare teams internationally, stating how physiotherapists, clinical exercise physiologists and personal trainers practice alongside each other in the United States of America. Similarly in Europe, physiotherapists, clinical exercise physiologists and kinesiotherapists cross-refer patients to one another.^[5]^ National studies^[12,13]^ have reported that such an approach to patient care has been linked to positive perceptions of healthcare management by patients^[12]^ and improved patient compliance with rehabilitation.^[13]^ Further South African-based research; however, has reported that OA is managed with a combination of conservative and surgical interventions that demonstrate considerable variations in costs, utilisation of interventions, and access to care.^[7,9,12]^ The varied nature of OA care^[14]^ is not only less effective for some patients, but potentially more expensive due to duplicate and sub-optimal treatment.^[8]^ Models that incorporate a team approach may mitigate these concerns, thereby facilitating improved patient outcomes and redistribution of OA management costs.^[8]^

The success of a team approach is dependent on positive perceptions among individual HCPs.^[15]^ These perceptions are formed by the HCP’s knowledge regarding each profession’s SoP.^[15]^ However, a lack of familiarity with the services offered by and the roles of various disciplines often leads to poor engagement and uptake of a team approach.^[5]^ A local study^[15]^ reported that the perceptions among various disciplines were mainly negative as a result of a lack of knowledge around other professions’ roles, thereby hindering a collaborative environment.^[15]^ An unawareness of other professions’ SoP may have implications for patient-focused care and may lead to grey areas in terms of rehabilitation jurisdiction^[5]^ and cross-referrals. Further contributing to the success of a team approach is effective communication among all the members of the healthcare team.^[16]^ Clear communication strategies will ensure the implementation of key components required for patient-focused care, such as well-ordered transitions between rehabilitation areas, sharing of relevant information and education, coordination and continuity of care, and outcomes that are important to the patient.^[16]^ Conversely, ineffective communication creates uneasiness, doubt and dissatisfaction with patient care, and has been linked to a lack of compliance with recommended treatment options by patients.^[17]^ Furthermore, ineffective communication has also been linked to increased stress, lack of job satisfaction and emotional burnout among HCPs.^[17]^

In order to foster a team environment, there needs to be clear communication, positive perceptions and a greater awareness among HCPs.^[12]^ Positive perceptions of different HCPs are developed during formal academic training through interprofessional education (IPE), thus creating an awareness and environment for organised healthcare teams.^[12,18]^ There are four key competencies that address collaboration within healthcare teams.^[18]^ These competencies include: values and ethics for collaborative practice, knowledge of one’s own role and responsibility and those of other professions, communication among patients, families, communities, and professionals, and teamwork.^[18]^ The emersion within these competencies has been identified as critical for the provision of efficient healthcare, given the complexity of patients’ healthcare needs and the range of HCPs involved.^[15]^

Numerous studies^[15,19,20]^ have explored the perceptions of various HCPs towards each other; however, no studies have described rehabilitative professionals’ views on a team approach to OA management in the South African healthcare setting. Furthermore, there is a dearth of published OA research in South Africa with identified gaps in the continuum of the care pathway for the management of OA.^[12]^ Therefore, the purpose of this study was to explore and describe the viewpoints of various biokineticists and physiotherapists regarding a team approach to OA management in the South African public and private healthcare setting. Awareness of the diverse and multidisciplinary nature of OA care and rehabilitative professionals’ views of a team approach to OA care could guide best practice recommendations and strategies to enhance organised teamwork to promote service delivery and quality person-focused care for the OA patient in South Africa.

## Methods

### Study design

A descriptive, cross-sectional survey research design was used in this study. The design utilised quantitative data collected initially to explore and describe the viewpoints of biokineticists and physiotherapists regarding a team approach to OA management.

### Participants

The target population consisted of biokineticists and physiotherapists who have worked with or are currently working with an OA patient population within either the public and/or private healthcare sectors. Gatekeepers from healthcare profession associations were approached, and permission to invite volunteers to be part of the study was granted. The associations included the Biokinetics Association of South Africa, the South African Society of Physiotherapy, and the South African Sports Medicine Association. Participants were recruited using a convenience sampling technique whereby the gatekeepers of the respective associations emailed the HCPs via their online practitioner database. The email included a description of the study and a link to the study’s online questionnaire. Within this description it stated that the study was specific to OA management and participants were required to have worked with or are currently working with such a patient population. Thereby, prospective participants were excluded if they were not involved in OA management, if they were not registered with the HPCSA, or if they were students or interns. Ethical clearance was provided by the Nelson Mandela University Research Ethics Committee: Human (H20-HEA-HMS-005).

### Data collection

Data were collected using a closed-ended online questionnaire (supplementary data). The questionnaire was designed by the principal investigator, guided by a group of multidisciplinary academics within the Faculty of Health Sciences, following a structured review of the literature. A panel of subject experts (including academics and HCPs) then reviewed and edited the questionnaire. The link for the online questionnaire was distributed via email by the gatekeepers of the associations to members of the statutory bodies. QuestionPro® was the software used to capture, distribute and analyse the descriptive data. All participants could complete the questionnaire anonymously. Thereby informed consent was provided by selecting the link to access the questionnaire. Questions were designed to prompt for the selection of answers from a list of options with some questions adopting a Likert-type scale. The questionnaire was circulated for a period of six months. The complete questionnaire that was circulated consisted of the following sections: (i) descriptive data in terms of the number of years in practice, from which healthcare sector participants practised, and their current practice setting; and (ii) participants’ ratings of various factors that influence team-based OA management. These factors were based on the IPE competencies, the questions included: to describe the communication between HCPs in the OA management team, identification of the knowledge of one’s own role and responsibility, and those of other members within the OA healthcare team, and finally, to explore their stance on interprofessional engagement and teamwork for OA management in South Africa.

### Statistical analysis

Quantitative, descriptive data were exported from QuestionPro® to Microsoft Excel spreadsheets for coding purposes. Statistical analysis was performed using the Statistical Package for Social Science (SPSS) version 26.0 (IBM Corp. 2019. IBM SPSS Statistics for Windows, Version 26.0. Armonk, NY: IBM Corp). Data were presented as frequencies and percentage distributions for categorical data. Cross tabulations, together with Chi-square analyses, were performed to quantitatively analyse the relationship between biokineticists and physiotherapists and their rating of a team approach in OA management. Significant results (p<0.05) were emphasised.

## Results

### Descriptive data

The descriptive data of the participating HCPs are shown in Table 1. The study cohort comprised of 212 participants of which 47 were biokineticists and 165 were physiotherapists. Data showed that biokineticists and physiotherapists differ with respect to the number of years they have been in practice (p=0.04). When evaluating the data distribution among the physiotherapist participants, an even distribution of years in practice was noted. However, among the cohort of biokineticists, 66% of participants indicated that they had been in practice for 10 years or less. Additionally, there is evidence that the biokineticists and physiotherapists differ with respect to which healthcare sector they practice from (p<0.001). Results show that the majority of the physiotherapists (82%) practiced solely in the private healthcare sector, with 12% in the public healthcare sector, whereas 66% of biokineticists practiced in private healthcare or a combination of private and public (19%) or private and corporate (9%) sectors. Table 1 further illustrates the current practice setting of biokineticists and physiotherapists. A number of physiotherapists described their practice setting within a hospital and/or clinic (32%) or as a solo practice (33%). Biokineticists strongly favoured solo practices (55%). It can be concluded that there is evidence that the biokineticists and physiotherapists differ with respect to their current practice set-up (p=0.002).

### Team approach in osteoarthritis management

Table 2 indicates biokineticists’ and physiotherapists’ rating of a team approach to OA management. There is no evidence that the biokineticists and physiotherapists differ with respect to how they rate the overall communication between team members (p=0.68). Communication was viewed as neither of a high nor low quality by biokineticists (43%) and physiotherapists (36%). Respectively, 54% and 69% of the biokineticists and physiotherapists felt adequately equipped on their understanding of the SoP of various healthcare professions involved in OA management. There is, however, no evidence that the biokineticists and physiotherapists differ with respect to how they rate their competence regarding their knowledge of the various SoPs (p=0.22). There is evidence, however, that the biokineticists and physiotherapists differ with respect to how they rate interprofessional engagement (p=0.003). Interprofessional engagement was viewed as extremely important among the study biokineticists (57%), whereas the physiotherapists rated interprofessional engagement very (56%) to somewhat important (12%) in the rehabilitation of an OA patient. There is no evidence of a difference between the exposure of IPE during the training of biokineticists and physiotherapists (p=0.61), with 43% of participating professionals indicating that they had not been exposed to IPE initiatives. Finally, the majority of the biokineticists (93%) and physiotherapists (91%) agreed that the South African healthcare system would benefit from structured healthcare teams.

## Discussion

The aim of this study was to explore and describe the viewpoints of biokineticists and physiotherapists regarding a team approach to OA management. Based on the IPE competencies required for effective teamwork in healthcare, the major findings of this study reported that communication in the OA management team was viewed as neither of a high nor low quality among HCPs. Furthermore, the participating biokineticists and physiotherapists felt adequately equipped on their understanding of the SoP of various healthcare professions involved in OA management; however, a large number of these professionals indicated that they had not been exposed to IPE initiatives.

The cohort of physiotherapists in this study reported a range of experience in practice, whereas, a larger number of biokineticists reported practicing for less than 10 years. Unlike physiotherapy, biokinetics is the latest addition to the rehabilitative professions group, and this descriptive finding can be supported by the gain in popularity in recent years and the subsequent recognition of the profession of biokinetics in the South African healthcare system.^[4,5]^ The majority of both professional groups reported practicing in the private healthcare sector. The demand for rehabilitative professions, such as biokinetics and physiotherapy in both the public and private healthcare sectors, is evident based on the rise of non-communicable diseases, including OA, in South Africa.^[1,6]^ The need for biokinetics in the public sector has received growing attention in recent years; however, lack of resources and funding for rehabilitation still proves to be considerable barriers.^[1]^

The effectiveness of healthcare teams globally has been highlighted in the literature,^[5,11]^ particularly in the management of OA as this condition is well suited to this model given the input required from multiple disciplines to meet the broad needs of these patients.^[8]^ A team approach focused on outcomes that matter to patients may optimise patient outcomes,^[8]^ improve perceptions of healthcare management by patients,^[12]^ increase patient compliance with rehabilitation,^[13]^ and redistribute interventional costs.^[8]^ There is, therefore, calls to establish these teams in South Africa.^[7,12,15]^ Nonetheless, the current study reported that solo practices were the favoured practice setting for both biokineticists and physiotherapists, with both profession groups further favouring partnership practices with a professional in the same discipline. Care provided by a single professional group may fail to address the complexity of OA patient needs,^[2]^ thereby being less successful and potentially more expensive because of unnecessary or inappropriate interventions.^[2,8,12]^ Poor patient outcomes could lead to decreased patient compliance with rehabilitation.^[13]^

Mlenzana and Frantz^[16]^ stated that the success of a team approach is dependent on effective communication among all members of the healthcare team. Effective communication was not found to be present in the results of the study as the data showed that communication was viewed as neither of a high nor low quality by a large number of rehabilitative professionals. Ineffective communication has been noted in the literature, especially regarding OA care,^[2,12]^ and has significant implications for patient outcomes.^[2,8,17]^ Numerous studies have demonstrated that better communication methods improve decision quality, confidence, satisfaction and compliance with intervention options, and professional patient engagement.^[8,17]^ Key aspects in the improvement of the practical nature of patient-focused care include information transfer between HCPs, shared decision-making^[18]^ and listening to the needs of the patient.^[2]^ These communication practicalities can be supported during the implementation phase by advocating for sufficient consultation time which allows the patient to feel heard by discussing their overall healthcare needs and actively engaging with their HCP regarding their treatment plan.^[2,12]^ Furthermore, virtual communication pathways, such as Telehealth and/or interoperable electronic health record systems, increase patients’ and professionals’ reachability and information transfer.^[2]^ Such virtual communication methods may prove valuable and consistent when used in conjunction with a communication framework established through IPE interventions which may enhance the capacity of HCPs to deliver improved coordinated care.^[2,18]^ Therefore, this output advocates for education initiatives for improved communication methods between members of the OA healthcare team.

In addition to effective communication, collaborative interactions stem from positive perceptions among HCPs.^[15,19,20]^ Positive perceptions are attained through an understanding of discipline-specific roles.^[15]^ The current study reported that the participating biokineticists and physiotherapists felt adequately equipped regarding their understanding of the SoP of various healthcare professions involved in OA management. A promising finding when compared to previous research which found no evidence of the knowledge that physiotherapists may have regarding biokineticists and other allied professionals.^[15]^ Moreover, this understanding of the SoP of the various healthcare disciplines encourages interprofessional engagement.^[5, 15]^ This study, however, suggests that while both biokineticists and physiotherapists identified the importance of interprofessional engagement, the study’s biokineticists were more in favour of interprofessional engagement when compared to the physiotherapists. Interestingly, a study by Ellapen et al.^[15]^ agrees by stating that biokineticists were favourably inclined to interprofessional engagement compared to physiotherapists, who were more apprehensive towards collaborative relations owing to their perception that these professionals were trespassing on their SoP. It is, therefore, important to address previous opinions of fragmented healthcare experiences among HCPs in order to strengthen both the therapeutic alliance and health outcomes for the patients.

Emphasis should be placed on this level of appreciation and awareness of other HCPs’ roles and responsibilities^[5]^ to facilitate team-based management, and thereby guide appropriate referral pathways and interventions. To accomplish this, literature has supported the notion that the implementation of IPE, as a vehicle for efficient collaborative engagement, should be promoted beyond a tertiary level^[15]^ and become an essential component of a healthcare team’s continuing professional training.^[18]^ Healthcare professional associations are perfectly positioned to begin instituting quarterly roadshows and workshops to encourage interprofessional collaboration and education strategies among HCPs in practice. This educational setting would shape team identity, add to relational therapy skills, increase the understanding of different rehabilitation phases, and reduce misperceptions of the different professions’ roles.^[18]^ The importance of IPE to team functioning and healthcare provision prompted the researchers to identify the exposure to IPE among the study’s participants. The results showed that a large number of the participating professionals had not been exposed to IPE during their training. To make better use of interventional costs,^[8]^ the establishment of appropriate referral pathways^[15]^ and collaborative relations, and to more effectively meet the complex needs of OA patients,^[2]^ it is essential that healthcare educational and organisational systems prioritise IPE initiatives.

### Limitations and recommendations for future research

While the cohort of participants was limited, future studies should aim at contributing to this research on a larger scale and elaborate on rehabilitative professionals as pivotal members of the healthcare team. That said, this study only included two rehabilitative profession groups, which is not wholly representative of a complete healthcare team. Additionally, the study sample excluded students and interns. Therefore the most current investigation of educational practices may have been beyond the scope of the study.

## Conclusion

The study showed that both professions were well-versed in the SoP of each of the members involved in the OA management team; however, communication among members was not optimal. The key IPE competencies for the promotion of a team approach to patient-focused care are stressed in the concluding remarks of this research. Interprofessional education is one way to improve the functioning of HCPs within a team and engagement with these competencies is critical to the provision of efficient healthcare. Therefore, while this study creates an awareness of the benefits of team-based management of OA, the findings could stimulate further debate on the optimal implementation of the key competencies required for effective communication and teamwork, thereby facilitating patient-focused care and the appropriate referral systems. Furthermore, this study aimed to contribute to the paucity of research concerning rehabilitative professionals’ views of a team approach and highlight the importance of their respective roles in OA management, which may contribute to the mutual appreciation of the different professions, thus preventing trespassing on the SoP of other professions and calling attention to the need for a team approach in clinical practice.

## Supplementary Information



## Figures and Tables

**Table 1:** Descriptive data of healthcare professionals (n=212)

	**Frequency (%)**			

**Profession**				
Physiotherapy	165 (78)			
Biokinetics	47 (22)			

	**Frequency (%)**		**Chi-square**	**p-value**

	**Physiotherapists**	**Biokineticists**		
	
**Number of years in practice**			8.18	0.04*
< 6 years	40 (24)	16 (34)		
6–10 years	40 (24)	15 (32)		
11–20 years	44 (27)	13 (28)		
>20 years	41 (25)	3 (6)		

**Healthcare sector currently practicing from**			18.02	<0.001*
Public sector	20 (12)	3 (6)		
Private sector	135 (82)	31 (66)		
Combination public and private sector	7 (4)	9 (19)		
Combination of private and corporate sector	3 (2)	4 (9)		

**Current practice setting**			14.82	0.002*
Solo practice	55 (33)	26 (55)		
Partnership practice with a practitioner in the same discipline as myself	43 (26)	11 (23)		
Partnership practice with a practitioner in a complementary discipline to myself	15 (9)	7 (15)		
Practice is within a hospital / clinic setting	52 (32)	3 (6)		

Data are expressed as n (%).

* indicates p<0.05.

**Table 2:** Rating team-based osteoarthritis management in a South African landscape

	**Frequency (%)**		**Chi-square**	**p-value**

	**Physiotherapists (n=163)**	**Biokineticists (n=47)**		
	
**Overall communication between team members**			2.30	0.68
Very high quality	13 (8)	6 (13)		
High quality	46 (28)	10 (21)		
Neither high nor low quality	59 (36)	20 (43)		
Low quality	35 (21)	9 (19)		
Very low quality	10 (6)	2 (4)		

**Knowledge of the scope of practice of various healthcare professions**			5.76	0.22
Extremely familiar	23 (14)	5 (11)		
Very familiar	90 (55)	20 (43)		
Somewhat familiar	40 (25)	17 (36)		
Not so familiar	8 (5)	5 (11)		
Not at all familiar	2 (1)	0 (0)		

**Importance of interprofessional engagement**			11.55	0.003*
Extremely important	55 (32)	27 (57)		
Very important	92 (56)	19 (40)		
Somewhat important	20 (12)	1 (2)		
Not so important	0 (0)	0 (0)		

**Exposure to interprofessional education**			0.26	0.61
Yes	93 (57)	25 (53)		
No	69 (43)	22 (47)		

**Stance on healthcare teams in a South African landscape**			2.91	0.41
Extremely important	105 (64)	35(74)		
Very important	44 (27)	9 (19)		
Somewhat important	11 (7)	3 (6)		
Not so important	5 (3)	0 (0)		

Data are expressed as n (%).

* indicates p<0.05.
