# Supplementary material for: Team approach to osteoarthritis management: Viewpoints of biokineticists and physiotherapists in South Africa
Source: S Afr J Sports Med. 2023 May 9;35(1):v35i1a15260. doi: 10.17159/2078-516X/2023/v35i1a15260 (PMC10798614; doi:10.17159/2078-516X/2023/v35i1a15260)
Supplement: Supplementary file 1 [file 2078-516X-35-v35i1a15260-s001.pdf]

Team approach to osteoarthritis management: Viewpoints of biokineticists and physiotherapists in South Africa

Supplementary document – study questionnaire

This is the descriptive version of the questionnaire used in the study. The questionnaire was transcribed onto the Nelson Mandela University recommended software QuestionsPro® for the purposes of circulating the questionnaire and collecting the responses.

Team approach to osteoarthritis management: Viewpoints of biokineticists and physiotherapists in South Africa

This questionnaire will consist of 12 questions divided into two sections:
Q1-Q7 Descriptive data
Q8-Q12 The multidisciplinary medical team in osteoarthritis rehabilitation / management

SECTION 1: DESCRIPTIVE DATA

1. Choose your profession:
   - Biokineticist
   - Physiotherapist
2. Select your gender:
   - Male
   - Female
3. Select your age category:
   - 18 to 24
   - 25 to 34
   - 35 to 44
   - 45 to 54
   - 55 to 64
   - 65 to 74
   - 75 or older
4. Choose the type of practice that best describes your current practice:
   - Solo practice
   - Partnership practice with a practitioner in the same discipline as myself
   - Partnership practice with a practitioner(s) in a complementary practice to myself
   - My practice is within a hospital / clinic setting
5. How long have you been in practice?
   - 0-5 years
   - 6-10 years
   - 11-20 years
   - >20 years
6. In which province are you currently practicing?
   - Western Cape
   - Eastern Cape
   - Northern Cape
   - North West
   - Free State
   - Kwazulu Natal
   - Gauteng
   - Limpopo
   - Mpumalanga
7. Is your practice aimed at the:
   - Public sector
   - Private sector
   - Corporate sector
   - Combination of private and public sector
   - Combination of private and corporate sector
   - Combination of public and corporate sector

SECTION 2: THE MULTIDISCIPLINARY MEDICAL TEAM IN OSTEOARTHRITIS REHABILITATION / MANAGEMENT

8. How would you rate the overall communication between a multidisciplinary osteoarthritis rehabilitation team?
    - Very high quality
    - High quality
    - Neither high nor low quality
    - Low quality
    - Very low quality

9. As a healthcare professional, do you feel adequately educated on other healthcare professionals' scope of practice in the rehabilitation of osteoarthritis?
    - Extremely familiar
    - Very familiar
    - Somewhat familiar
    - Not so familiar
    - Not at all familiar

10. Do you consider interprofessional engagement important in the rehabilitation of an osteoarthritic patient?
    - Extremely important
    - Very important
    - Somewhat important
    - Not so important
    - Not at all important

11. Have you been exposed to interprofessional education (IPE) during your training?
    - Yes
    - No

12. Do you think the South African healthcare system would benefit from structured multidisciplinary rehabilitation teams?
    - A great deal
    - A lot
    - A moderate amount
    - A little
    - Not at all

Square bullets mean that you can choose more than one option and the circle bullets mean that you can only choose one option.
